# ABHD5 stimulates PNPLA1-mediated ω-*O*-acylceramide biosynthesis essential for a functional skin permeability barrier[S]

**DOI:** 10.1194/jlr.M089771

**Published:** 2018-10-25

**Authors:** Benedikt Kien, Susanne Grond, Guenter Haemmerle, Achim Lass, Thomas O. Eichmann, Franz P. W. Radner

**Affiliations:** Institute of Molecular Biosciences,* University of Graz, Graz, Austria; Center for Explorative Lipidomics,§ Graz, Austria; BioTechMed-Graz,† Graz, Austria

**Keywords:** α/β-hydrolase domain-containing 5, patatin-like phospholipase domain-containing 1, ceramides, disease, epidermis, fatty acid, transferase, lipid biochemistry, ichthyosis, adipose triglyceride lipase, comparative gene identification 58

## Abstract

Mutations in the genes coding for patatin-like phospholipase domain-containing 1 (PNPLA1) and α/β-hydrolase domain-containing 5 (ABHD5), also known as comparative gene identification 58, are causative for ichthyosis, a severe skin barrier disorder. Individuals with mutations in either of these genes show a defect in epidermal ω-*O*-acylceramide (AcylCer) biosynthesis, suggesting that PNPLA1 and ABHD5 act in the same metabolic pathway. In this report, we identified ABHD5 as a coactivator of PNPLA1 that stimulates the esterification of ω-hydroxy ceramides with linoleic acid for AcylCer biosynthesis. ABHD5 interacts with PNPLA1 and recruits the enzyme to its putative triacylglycerol substrate onto cytosolic lipid droplets. Conversely, alleles of *ABHD5* carrying point mutations associated with ichthyosis in humans failed to accelerate PNPLA1-mediated AcylCer biosynthesis. Our findings establish an important biochemical function of ABHD5 in interacting with PNPLA1 to synthesize crucial epidermal lipids, emphasizing the significance of these proteins in the formation of a functional skin permeability barrier.

The skin forms a permeability barrier between the external environment and the individual’s internal milieu, thereby protecting the individual from invasion of pathogens and harmful substances such as chemicals and allergens as well as from the loss of water and electrolytes (1, 2). This barrier resides in the stratum corneum (SC), the outermost layer of the epidermis, and consists of terminally differentiated keratinocytes (corneocytes) that are embedded in a lipid-enriched matrix consisting of ceramides, cholesterol, and nonesterified FAs (3, 4). To provide permeability barrier function, the extracellular lipids form lamellar membranes in the SC, which requires a scaffold on the external surface of the corneocytes consisting of protein-bound ultra-long-chain (ULC) ω-hydroxy ceramides known as the corneocyte-bound lipid envelope (CLE) (5). The formation of this hydrophobic lipid monolayer largely depends on the presence of ω-*O*-acylceramides (AcylCers) in the SC. The biosynthesis of these hydrophobic barrier lipids occurs in the stratum granulosum by condensation of three different carbon chains, a long-chain base (mainly sphingosine), an ω-hydroxylated ULC FA (28–36 carbon atoms), and linoleic acid (**Fig. 1A**), and requires the action of at least five different enzymes (Fig. 1B) (6, 7). For CLE formation, AcylCers are transferred to the external SC where the ω-esterified linoleoyl moiety is further processed, resulting in ULC ω-hydroxy ceramides covalently bound to specific amino acid residues of structural proteins of the corneocytes (8, 9).

**Fig. 1.**
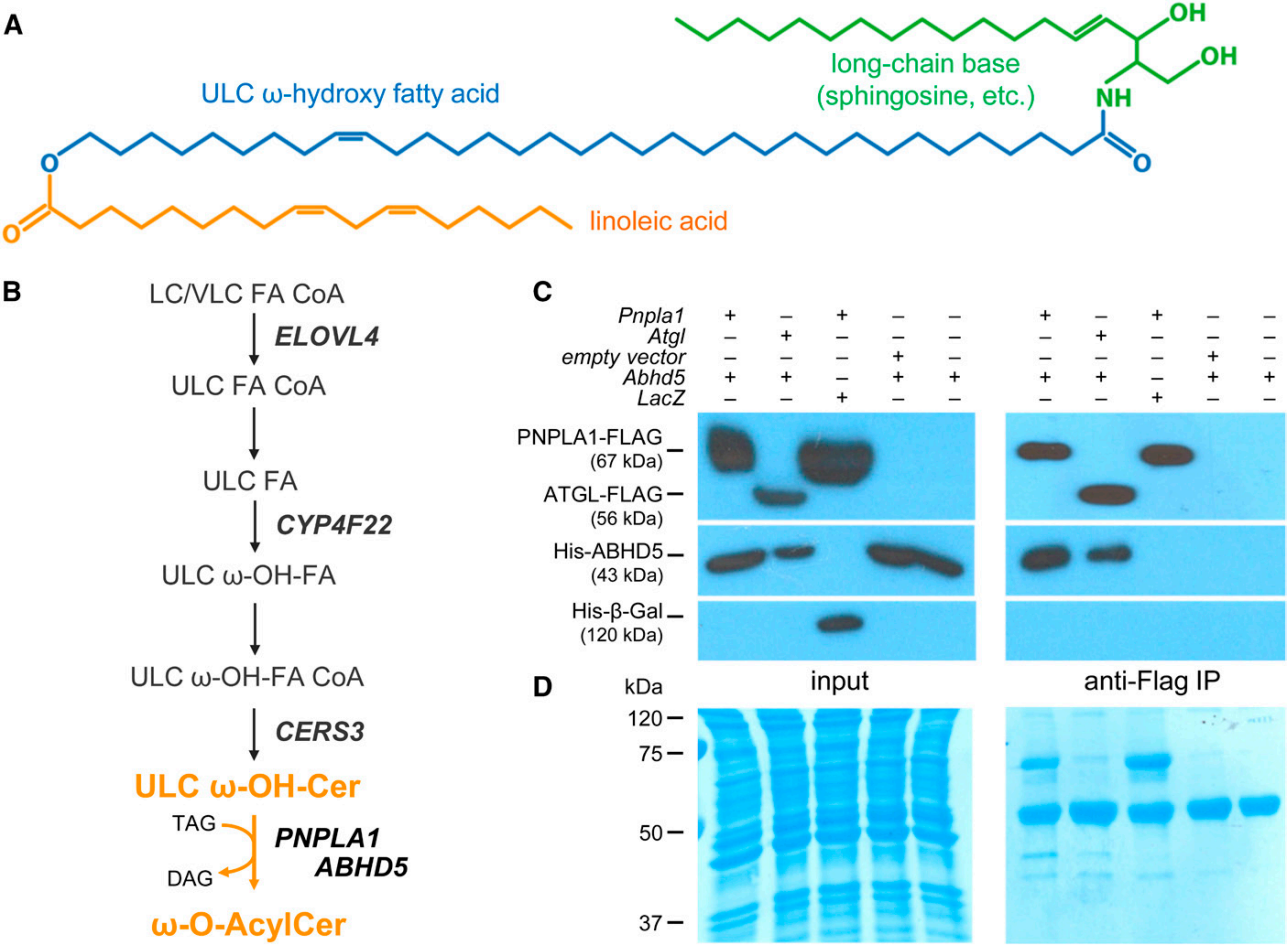
Protein interaction of ABHD5 with PNPLA1 indicates a direct role of ABHD5 in AcylCer biosynthesis. A: Molecular structure of AcylCer. B: Proposed pathway of AcylCer biosynthesis. Transacylation of linoleic acid from TAG molecules onto ULC ω-hydroxy ceramides requires the presence of PNPLA1 and ABHD5. C: His-tagged ABHD5 coimmunoprecipitates with FLAG-tagged PNPLA1. Recombinant proteins were detected in cell homogenates (input) and anti-FLAG IPs by immunoblot analysis using adequate antibodies. D: Coomassie blue staining indicates equal protein loading. Cer, ceramide; DAG, diacylglycerol; FA, fatty acid; IP, immunoprecipitate; LC, long chain; VL, very long chain; ω-OH, omega-hydroxy.

Mutations in genes that are involved in either the synthesis, secretion, or processing of barrier lipids lead to a defective skin permeability barrier and concomitantly to the pathophysiology of ichthyosis (10, 11). This rare scaling disorder is characterized by epidermal hyperplasia that results in the formation of multiple layers of corneocytes (hyperkeratosis) and abnormal desquamation leading to the detachment of large SC flakes (10–14). Although ichthyoses always display this visible scaling, they are phenotypically and genetically extremely heterogeneous. Since the first ichthyosis consensus conference (11), the various forms of the disease are now classified into nonsyndromic ichthyoses, in which the disease is limited to the skin, and syndromic ichthyoses, where the disorder additionally affects other tissues and organs.

Humans carrying mutant alleles of the lipolytic coactivator α/β-hydrolase domain-containing 5 (ABHD5), also designated as comparative gene identification 58, develop neutral lipid storage disease with ichthyosis [NLSDI; also referred to as Chanarin-Dorfman syndrome (OMIM: 275630)] (15, 16). This rare genetic disorder belongs to the subgroup of syndromic ichthyoses that also affect lipid and energy homeostasis in the liver and brain. In mice, the phenotype of ABHD5 deficiency is even more severe, leading to premature death soon after birth due to increased transepidermal water loss and desiccation (17, 18). ABHD5 is known as a coactivating protein required for the stimulation of the hydrolytic activity of adipose triglyceride lipase (ATGL; also known as patatin-like phospholipase domain-containing 2). ATGL is a key enzyme in the catabolism of intracellular, nonlysosomal triacylglycerol (TAG) depositions that are present in virtually all tissues of the body (19, 20). Remarkably, humans and mice with mutant *ATGL* alleles also suffer from neutral lipid storage disease (OMIM: 610717) but show normal skin development and function, which indicates that ABHD5 exerts an ATGL-independent role in skin physiology (17, 21). Functional analyses of ABHD5-deficient skin from mice and humans revealed a lack of AcylCer synthesis, resulting in defective CLE formation (17, 18, 22). In parallel, mutant skin accumulated free extractable ULC ω-hydroxy ceramides, the precursor lipids for AcylCer formation, which indicates a role of ABHD5 in the final step of AcylCer biosynthesis (Fig. 1B) (18).

Important progress on elucidating the ATGL-independent function of ABHD5 in epidermal lipid metabolism and skin barrier formation was made when we identified mutations in the ATGL-homologous gene patatin-like phospholipase domain-containing 1 (*PNPLA1*) that were linked to ichthyosis development in humans and golden retriever dogs (23). Functional analyses of affected skin demonstrated that humans and mice with defective PNPLA1 function develop an identical defect in AcylCer biosynthesis, as shown for ABHD5 deficiency (24–26). *PNPLA1* mutant epidermis lacked AcylCer and accumulated ULC ω-hydroxy ceramides, leading to a severe dysfunction of the skin permeability barrier in affected individuals. In line with these observations, PNPLA1 has recently been suggested to act as transacylase, utilizing TAG as an acyl donor for the synthesis of AcylCer (27). However, the measured transacylase activity of PNPLA1 was relatively low in in vitro assays, suggesting that PNPLA1 requires additional proteins to reach full enzymatic activity similar to what has been demonstrated for the homologous protein ATGL that is activated by ABHD5. Here, we show that ABHD5 interacts with PNPLA1 and stimulates AcylCer biosynthesis.

## MATERIALS AND METHODS

### cDNA cloning of recombinant proteins

Human and murine cDNAs were prepared from total RNA obtained from murine epidermis or white adipose tissue or from in vitro differentiated human primary keratinocytes as described previously (28) using SuperScript Reverse Transcriptase protocol (Invitrogen Life Technologies, Carlsbad, CA). Sequences containing the complete open-reading frame of human *PNPLA1*, FA elongase-4 (*ELOVL4*), cytochrome P450 family 4 subfamily F member 22 (*CYP4F22*), ceramide synthase 3 (*CERS3*), and murine *Pnpla1*, *Abhd5*, and *Atgl* were amplified by PCR from respective cDNA using Phusion High-Fidelity DNA Polymerase (Finnzymes, Espoo, Finland) and the gene-specific primer pairs listed in supplemental Table S1. PCR amplification products were ligated to compatible restriction sites of the following eukaryotic expression vectors: pcDNA4/HisMax (Invitrogen Life Technologies), pFLAG-CMV-5.1 (Sigma-Aldrich, St Louis, MO), pEYFP-C1 (Takara Bio USA, Mountain View, CA), pECFP-C1 (Takara Bio USA), or pLVX-ultra-IRES-Puro (see below). Human *ABHD5*, murine *Abhd5*, and *Pnpla1* were cloned into pcDNA4/HisMax vector as described previously (19, 23). A control pcDNA4/HisMax vector expressing β-galactosidase (β-gal) was provided by the manufacturer (Invitrogen Life Technologies). The point mutations in *ABHD5*, c.389A>C (p.Gln130Pro), c.778G>A (p.Glu260Lys), and c.568C>T (p.Gln190Ter), were generated as described previously (19). Sequence analyses were performed at Microsynth AG (Balgach, Switzerland).

### Cell culture and transfection

HEK 293T cells (ATCC CRL-3216) or COS-7 cells (ATCC CRL-1651) were cultivated in DMEM (Thermo Fisher Scientific, Waltham, MA) supplemented with 10% FBS (Sigma-Aldrich) on cell culture dishes precoated with 0.1% gelatin (Sigma-Aldrich) in a standard humidified 7% CO_2_ atmosphere at 37°C. Cells were transfected with 2.5 µg plasmid DNA using Metafectene (Biontex GmbH, Munich, Germany) according to the manufacturer’s instructions.

### Cell-based AcylCer synthesis assay

HEK 293T cells were transfected with plasmids as indicated. Twenty-four hours after transfection, culture medium was changed to DMEM without FBS supplementation. After 30 min of incubation, cells were cultivated in DMEM supplemented with 20 µM nervonic acid, 10 µM linoleic acid, 2 µM sphingosine, and 10 µM [1-^14^C]linoleic acid (ARC 0294; American Radiolabeled Chemicals, St. Louis, MO) or 25 nM [3-^3^H]sphingosine (ART 0490; American Radiolabeled Chemicals) at 37°C for 6 h. FAs were complexed to essentially FA-free BSA (Sigma-Aldrich) at a molar FA-BSA ratio of 3:1. After washing cells twice with PBS, lipids were extracted three times with chloroform-methanol-glacial acetic acid (66/33/1; v/v/v). Phase separation was obtained by the addition of 1/5 vol. water and centrifugation at 1,400 *g* at room temperature for 5 min. The lower organic phase containing the lipids was collected and dried under a stream of nitrogen. Remaining cellular proteins were solubilized in 0.3 N NaOH and 0.1% SDS at 65°C overnight, and protein content was determined using Pierce BCA reagent (Thermo Fisher Scientific) and BSA as a standard. Lipids corresponding to equal amounts of protein were separated by normal-phase TLC as described previously (24). Radiolabeled lipids on TLC plates were detected by spraying a fluorographic reagent containing scintillation cocktail (Rotiszint; Carl Roth GmbH, Karlsruhe, Germany), methanol, and water (4/1/1; v/v/v), followed by exposure to a light-sensitive film (Amersham Hyperfilm ECL; GE Healthcare, Chicago, IL) at −80°C for 24–48 h. For the identification of the lipid spots corresponding to AcylCer, we radiolabeled lipid extracts from wild-type and PNPLA1-deficient epidermis (the latter of which is known to lack AcylCer) with [1-^14^C]linoleic acid as described previously (24) and used them as lipid standards.

### In vitro AcylCer synthesis assay

To obtain homogenates containing radiolabeled linoleic acid donor and ULC ω-hydroxy ceramide acceptor substrate for in vitro AcylCer synthesis assays, HEK 293T cells were transfected with a mammalian expression plasmid encoding *ELOVL4*, *CYP4F22*, and *CERS3* and labeled with [1-^14^C]linoleic acid as described above. In parallel, cells were transfected with a PNPLA1 or ABHD5 expression plasmid but were not incubated with radiolabeled linoleic acid. After 24 h, cells were scraped off culture dishes, washed three times with PBS, and disrupted in buffer A (0.25 M sucrose, 1 mM EDTA, 1 mM dithiothreitol, 20 mg/ml leupetine, 2 mg/ml antipain, 1 mg/ml pepstatin, pH 7.0) by sonication (Virsonic 475; Virtis, Gardiner, NJ). After the removal of nuclei and unbroken cells by centrifugation at 1,000 *g* and 4°C for 5 min, the protein concentration of cell homogenates was determined using Bradford reagent (Bio-Rad, Hercules, CA) and BSA as a standard. To determine the in vitro AcylCer synthesis activity of PNPLA1 and ABHD5, 100 µg protein of respective cell homogenates were mixed with equal amounts of homogenates containing lipid acceptor and radiolabeled linoleic acid donor substrate in a total assay buffer volume of 240 µl (50 mM HEPES/NaOH pH 7.4, 150 mM NaCl, 10% glycerol). After incubation in a water bath at 37°C for 60 min, the reaction was stopped by adding 1.2 ml chloroform-methanol-glacial acetic acid (66/33/1; v/v/v). The organic phase containing the lipids was obtained by centrifugation at 1,400 *g* at room temperature for 5 min. The synthesis of radiolabeled AcylCer was analyzed by TLC as described above.

### Coimmunoprecipitation experiment

HEK 293T cells were transfected with FLAG-PNPLA1 or FLAG-ATGL together with histidine (His)-ABHD5 or His-β-Gal expressing vectors using Metafectene as described above. Twenty-four hours after transfection, cells were harvested and lysed in lysis buffer (50 mM Tris/HCl pH 7.4, 150 mM NaCl, 1 mM EDTA, 1% NP-40, 20 μg/ml leupeptine, 2 μg/ml antipain, 1 μg/ml pepstatin) by incubation for 30 min at 4°C on a rotating wheel. Lysates were centrifuged at 1,000 *g* and 4°C for 5 min. Equal amounts of lysate proteins (1 mg) were incubated with 20 μl anti-FLAG M2-agarose beads (Sigma-Aldrich) overnight at 4°C on a rotating wheel. Thereafter, beads were extensively washed with lysis buffer and heated in 2× Laemmli buffer at 95°C for 10 min. Input and immunoprecipitation fractions were subjected to immunoblot analyses using appropriate antibodies.

### Confocal fluorescence microscopy

For localization studies, COS-7 cells were seeded in chambers mounted onto coverslips (Sarstedt, Nümbrecht, Germany) and transfected with yellow fluorescent protein (YFP)-tagged murine PNPLA1 and cyan fluorescent protein (CFP)-tagged murine ABHD5 expressing vectors using Metafectene as described above. Four hours after transfection, the culture medium was supplemented with 400 µM oleic acid complexed to essentially FA-free BSA (Sigma-Aldrich) at a molar FA-BSA ratio of 3:1 to promote lipid droplet formation. After 18 h, cells were imaged using a Leica SP5 confocal microscope equipped with a Leica HCX 63× 1.4 numerical aperture oil immersion objective. CFP and YFP fluorescence were excited at 458 and 514 nm, respectively, and emission was detected between 477 and 535 nm.

### Immunoblot analysis

Immunoblot analyses were performed according to standard protocols using anti-FLAG M2-HRP (A8592; Sigma-Aldrich) antibody or anti-His (27-4710-01; GE Healthcare), anti-Xpress (R910-25; Invitrogen) or anti-GAPDH primary antibody (2118S; Cell Signaling Technology, Danvers, MA, USA), and the appropriate HRP-conjugated secondary antibody. HRP-conjugated IgG was detected using Clarity Western ECL Substrate (Bio-Rad). Finally, membrane-bound proteins were stained with Coomassie blue staining solution [45% (v/v) ethanol, 10% (v/v) acetic acid, 0.25% (v/w) Coomassie Brilliant Blue R250] followed by destaining with a solution consisting of 30% (v/v) ethanol and 10% (v/v) acetic acid.

## RESULTS

### ABHD5 interacts with PNPLA1

To evaluate whether PNPLA1 is a potential interaction partner of ABHD5 similar to what has been previously reported for ATGL (19), we performed coimmunoprecipitation experiments with HEK 293T cells coexpressing murine FLAG-tagged PNPLA1 and murine His-tagged ABHD5 (Fig. 1C, D). Using this experimental setup, we observed that ABHD5 coimmunoprecipitated with FLAG-tagged PNPLA1 bound to anti-FLAG-tagged antibody-coated agarose beads. Similarly, His-tagged ABHD5 coimmunoprecipitated with FLAG-tagged ATGL, which was used as a positive control in this assay. Experimental controls confirmed a specific interaction between PNPLA1 and ABHD5. We did not observe an unspecific binding between the FLAG- and the His-tag (controls: PNPLA1-FLAG, His-β-Gal; His-ABHD5, empty FLAG vector) or binding of His-tagged ABHD5 to the anti-FLAG-tagged antibody-coupled agarose beads (control: His-ABHD5 alone). This finding demonstrates that PNPLA1 interacts with ABHD5, suggesting a direct role of ABHD5 in AcylCer biosynthesis (18).

### ABHD5 recruits PNPLA1 to intracellular lipid droplets

**Fig. 2.**
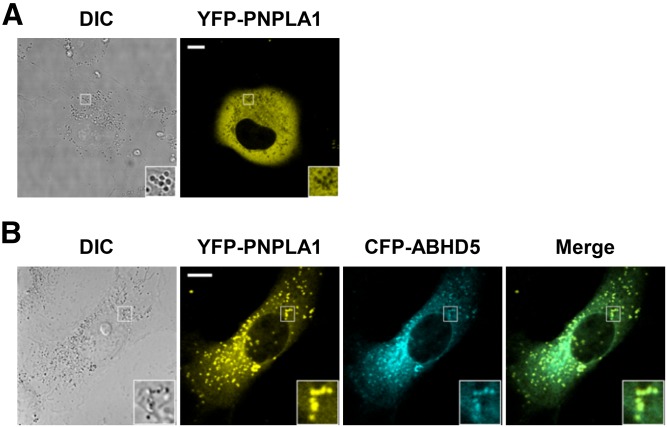
ABHD5 recruits PNPLA1 to lipid droplets. The intracellular localization of fluorescent-labeled proteins was analyzed by confocal laser-scanning microscopy in transfected COS-7 cells incubated with oleic acid to increase lipid droplet formation. A: Cells expressing YFP-PNPLA1 alone show cytoplasmic localization of the YFP signal. B: In the presence of CFP-ABHD5 expression, YFP-PNPLA1 colocalizes with ABHD5 at lipid droplets. Representative images are depicted. Scale bars, 10 µm. Insets, 3× magnification. DIC, differential interference contrast.

To investigate the protein interaction of PNPLA1 with ABHD5, we analyzed the intracellular localization of murine YFP-tagged PNPLA1 and CFP-tagged ABHD5 by live-cell imaging. Consistent with previous reports (29), fluorescent microscopy of YFP-PNPLA1-transfected COS-7 cells revealed an evenly distributed YFP signal, consistent with a cytosolic localization of the YFP-PNPLA1 fusion protein (**Fig. 2A**). Upon coexpression of YFP-PNPLA1 and CFP-ABHD5, we observed that both proteins colocalized at lipid droplets (Fig. 2B, merge image and inset), appearing as vesicular structures by differential interference contrast microscopy (Fig. 2B, differential interference contrast image and inset). Given that ABHD5 is a bona fide lipid droplet-associated protein (30, 31) (supplemental Fig. S1), our finding demonstrates that ABHD5 also interacts with PNPLA1 in a cellular system, thereby recruiting PNPLA1 to lipid droplets and facilitating the accessibility of PNPLA1’s proposed TAG acyl-donor substrate (27).

### Synthesis of ULC ω-hydroxy ceramides requires the presence of ELOVL4, CYP4F22, and CERS3 enzymatic activity

The identification and analysis of enzymes involved in AcylCer biosynthesis using a cell-based assay have been unsuccessful for a long time because most mammalian cell lines do not produce ULC ω-hydroxy ceramides, the precursors of AcylCer. Therefore, we genetically modified HEK 293T cells by transfection with a vector expressing a multicistronic mRNA transcript that encodes the three enzymes essential for ULC ω-hydroxy ceramide synthesis: ELOVL4, FA ω-hydroxylase CYP4F22, and CERS3 (Fig. 1B). In contrast to previous attempts to transfect cells with three separate expression plasmids singly encoding respective enzymes (32), our vector construct ensured ELOVL4, CYP4F22, and CERS3 expression simultaneously in each single cell to facilitate robust synthesis of ULC ω-hydroxy ceramides.

**Fig. 3.**
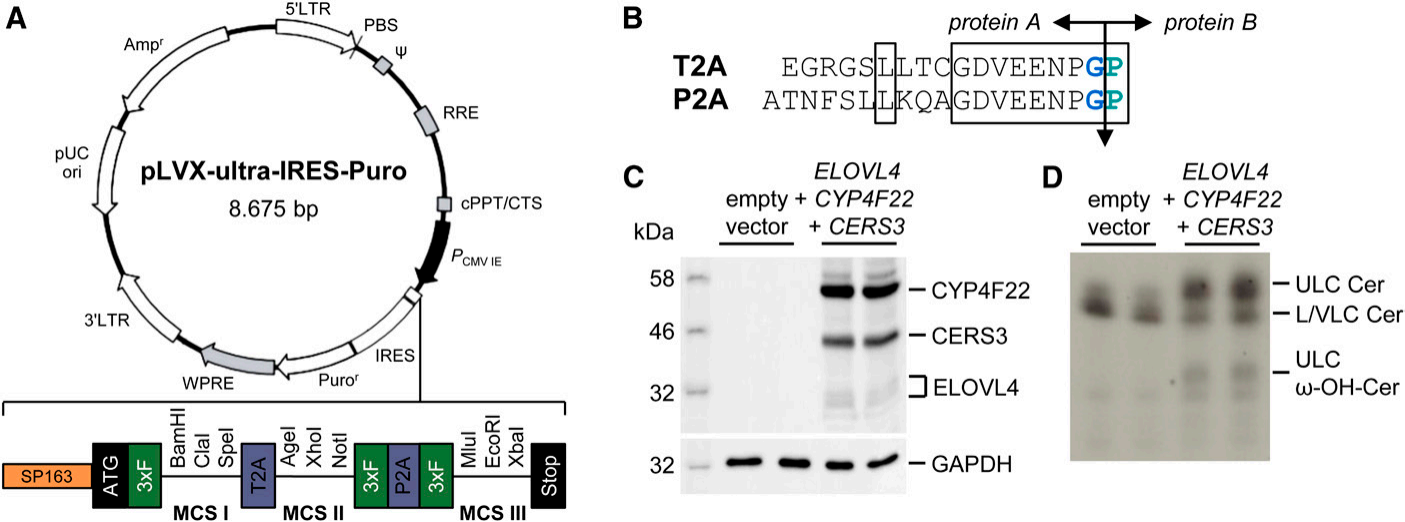
Expression of ELOVL4, CYP4F22, and CERS3 in HEK 293T cells leads to the synthesis of ULC ω-hydroxy ceramides. A: Vector map of the lentiviral multicistronic mammalian expression vector pLVX-ultra-IRES-Puro. B: Amino acid sequence of 2A regions from *Thosea asigna* virus (T2A) and porcine teschovirus-1 (P2A). Evolutionary conserved residues are boxed. The cleavage point between protein A and protein B is indicated by an arrow. C: Immunodetection of simultaneous expression of ELOVL4, CYP4F22, and CERS3 in HEK 293T cells using anti-FLAG M2-HRP antibody. GAPDH was used as a loading control. D: Analysis of sphingolipid synthesis in HEK 293T cells by TLC using [3-^3^H]sphingosine as a tracer. Data are representative of three independent experiments. Cer, ceramide; LC, long chain; VLC, very long chain; ω-OH, omega-hydroxy.

For the generation of the multicistronic expression vector pLVX-ultra-IRES-Puro, we inserted a de novo-synthesized DNA construct (Eurofins Genomics, Ebersberg, Germany) that encompassed a translational enhancer sequence and three different multiple cloning sites (MCSs) that are flanked by triple FLAG tags and separated by two different 2A peptide sequences from picornaviruses (33) into the MCS of the lentiviral vector pLVX-IRES-Puro (Takara Bio USA) (**Fig. 3A**). The coding sequences of *CERS3*, *CYP4F22*, and *ELOVL4* were then ligated into respective MCSs, and the resulting plasmid was transferred into HEK 293T cells. During protein translation of the mRNA fusion transcript, ribosomes cannot form peptide bonds between the conserved C-terminal glycine and proline of the 2A peptide sequences (Fig. 3B), which resulted in the expression of three separated proteins (Fig. 3C).

To analyze sphingolipid metabolism in this system, we cultivated transfected cells in medium containing [3-^3^H]sphingosine as a tracer and separated the extracted cellular lipids by TLC. Under these experimental conditions, we detected the production of ULC ω-hydroxy ceramides in cells with simultaneous expression of *ELOVL4*, *CYP4F22*, and *CERS3* but not in cells that were transfected with the empty vector control (Fig. 3D). These findings are consistent with data recently reported by Ohno et al. (32) and emphasize the significance of ELOVL4, CYP4F22, and CERS3 enzymatic activity for ULC ω-hydroxy ceramide biosynthesis (Fig. 1B). Accordingly, humans carrying mutant alleles of *ELOVL4*, *CYP4F22*, or *CERS3* develop a similar ichthyosis pathology as reported for ABHD5 and PNPLA1 deficiency due to the lack of ULC ω-hydroxy ceramides and AcylCer in the epidermis. The disturbed sphingolipid metabolism in these patients consequently results in a severe defect in CLE formation and skin barrier function (28, 34–36).

### ABHD5 stimulates PNPLA1-dependent ω-*O*-acylceramides synthesis

**Fig. 4.**
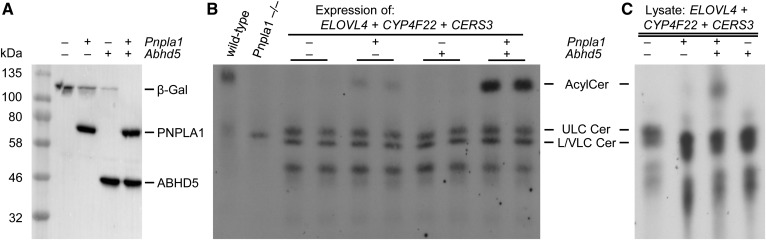
ABHD5 stimulates AcylCer production in the presence of PNPLA1. A: Immunodetection of murine PNPLA1 and ABHD5 expression in ULC ω-hydroxy ceramide-producing HEK 293T cells using anti-Xpress antibody. B: Analysis of AcylCer synthesis in HEK 293T cells by TLC using [1-^14^C]linoleic acid as a tracer. Radiolabeled epidermal lipid extracts from wild-type and *Pnpla1*^−/−^ mice were used as lipid standards for the identification of AcylCer spots. C: TLC analysis of in vitro AcylCer synthesis activity of indicated cell homogenates incubated with homogenates from [1-^14^C]linoleic acid-incubated HEK 293T cells that produce ULC ω-hydroxy ceramides by the expression of *ELOVL4*, *CYP4F22*, and *CERS3*. Data are representative of three independent experiments. Cer, ceramide; LC, long chain; VLC, very long chain.

ABHD5 has been previously shown to interact with ATGL, thereby stimulating its TAG hydrolase activity several-fold (19). These findings established an important biochemical function for ABHD5 in the lipolytic degradation of cellular TAG stores by ATGL. To examine whether ABDH5 also acts as a coactivator for PNPLA1 in stimulating the esterification of ULC ω-hydroxy ceramides with linoleic acid for AcylCer biosynthesis (Fig. 1B), we expressed respective murine proteins in our cell-based assay using appropriate expression plasmids (**Fig. 4A**). [1-^14^C]linoleic acid labeling subsequently revealed that cells expressing PNPLA1 produced detectable amounts of AcylCer compared with cells expressing β-Gal negative control (Fig. 4B). This finding is consistent with reported data (27) and corroborates that PNPLA1 catalyzes the final step in AcylCer biosynthesis. In contrast to PNPLA1, ABHD5 lacks AcylCer synthesis activity (27). Accordingly, ABHD5 expression in HEK 293T cells did not result in AcylCer production. Remarkably, when PNPLA1 and ABHD5 were coexpressed, we observed a drastic increase in AcylCer synthesis (Fig. 4B). Densitometry analysis of autoradiography signals demonstrated that the AcylCer level of cells expressing both PNPLA1 and ABHD5 increased more than 10-fold compared with cells expressing PNPLA1 alone (supplemental Fig. S2), demonstrating that ABHD5 stimulates PNPLA1-mediated AcylCer biosynthesis.

To additionally analyze AcylCer production in vitro, we prepared homogenates from [1-^14^C]linoleic acid-incubated HEK 293T cells that synthesized ULC ω-hydroxy ceramides due to the expression of recombinant ELOVL4, CYP4F22, and CERS3. Incubation of these radiolabeled homogenates with homogenates from nonlabeled cells expressing PNPLA1 resulted in the formation of detectable amounts of AcylCer, which was strongly induced by the additional presence of ABHD5 (Fig. 4C). In summary, these data provide compelling evidence that ABHD5 acts as an essential component of the AcylCer biosynthesis pathway by accelerating PNPLA1-dependent esterification of ULC ω-hydroxy ceramides.

### Point-mutated *ABHD5* alleles as present in ichthyosis patients fail to activate PNPLA1

**Fig. 5.**
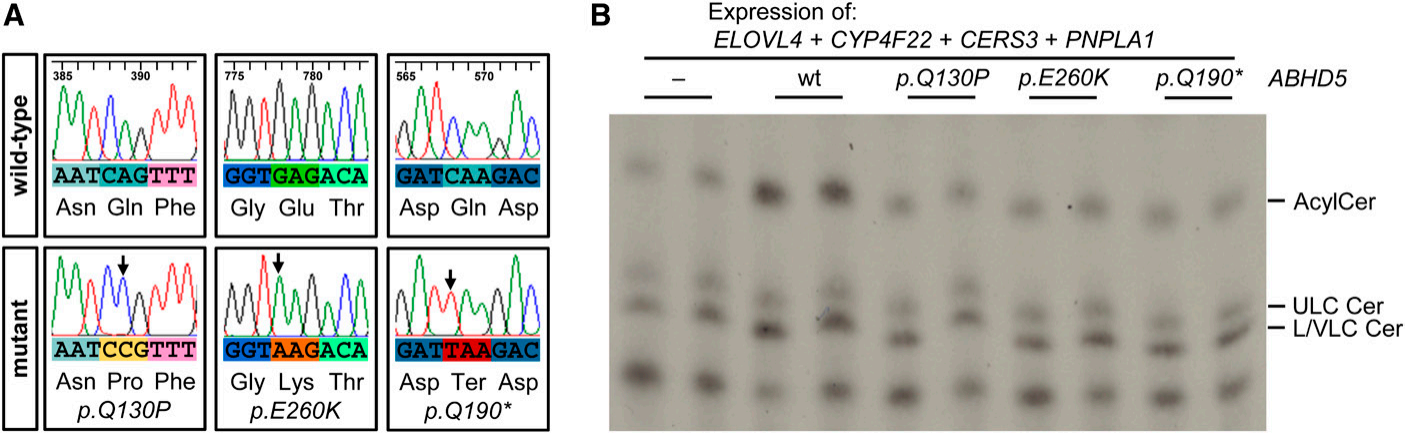
Point-mutated *ABHD5* alleles as present in ichthyosis patients fail to stimulate AcylCer biosynthesis. A: Sequencing analysis of *ABHD5* mutants. Point mutations are indicated by arrows. B: Analysis of AcylCer synthesis in HEK 293T cells in the presence of mutated ABHD5 proteins by TLC using [1-^14^C]linoleic acid as a tracer. Data are representative of three independent experiments. Cer, ceramide; LC, long chain; VLC, very long chain.

Alleles of *ABHD5* carrying point mutations associated with NLSDI have been reported to lose their ability to activate ATGL (19). Although this important finding provided a plausible biochemical explanation for the multisystemic TAG accumulation in affected patients, the exact molecular mechanism leading to the pathogenesis of ichthyosis remained unclear (16). To investigate the impact of *ABHD5* point mutations onto PNPLA1-mediated AcylCer formation, we selected one nonsense mutation and two missense mutations that have been reported to fail in activating ATGL (19). Point mutations were inserted into the coding sequence of wild-type *ABHD5* by site-directed mutagenesis, resulting in a premature stop codon (p.Gln190Ter) or a single amino acid substitution (p.Gln130Pro or p.Glu260Lys) in the protein (**Fig. 5A**). We then expressed human PNPLA1 together with each of these mutant variants of ABHD5 at comparable expression levels to wild-type protein in HEK 293T cells producing ULC ω-hydroxy ceramides (supplemental Fig. S3A, B). Similar to what we demonstrated for murine proteins, incubation of transfected cells with [1-^14^C]linoleic acid revealed that human wild-type ABHD5 increases cellular AcylCer levels in the presence of human PNPLA1 (Fig. 5B). In contrast, all analyzed mutant forms of ABHD5 were unable to stimulate PNPLA1-mediated AcylCer biosynthesis. AcylCer levels in these cells were comparable to those in cells expressing PNPLA1 alone (Fig. 5B). Based on our results, we conclude that mutations in human *ABHD5* associated with NLSDI result in a loss of the PNPLA1-activating function of ABHD5.

## DISCUSSION

In this report, we identified the long-sought ATGL-independent function of ABHD5 in epidermal lipid metabolism. Our data demonstrate that ABHD5 interacts with PNPLA1 and targets the enzyme onto lipid droplets. This process is assumed to facilitate the access of PNPLA1 to its putative TAG acyl-donor substrate, thereby stimulating the AcylCer biosynthesis required for a functional skin permeability barrier. Accordingly, ABHD5 does not only serve as a coactivator for ATGL to degrade cellular TAG stores but also is critical for PNPLA1 function in regulating skin lipid homeostasis.

To date, several studies have demonstrated an interaction of ABHD5 with proteins involved in cellular lipid and energy metabolism (37). One of the best characterized interactions is the activation of ATGL by ABHD5 that has been identified in our laboratory (19). Although the exact mechanism of ATGL activation is still elusive, data from other well-known lipases suggest that coactivator binding increases the hydrophobicity of the heterodimeric complex, which promotes enhanced substrate binding and more efficient enzymatic activity at the water-lipid interphase (38, 39). In accordance with these observations, ABHD5 has recently been reported to recruit the adipose FA binding protein to the lipid droplet surface for the binding of FA, the lipolytic product generated by ATGL (40). This process of FA capture in the nascent state prevents product inhibition of ATGL-mediated lipolysis and probably affects the association of the enzyme with lipid droplets (41). Considering the close evolutionary relationship between PNPLA1 and ATGL, it is conceivable that the interaction of ABHD5 with PNPLA1 affects the supply of acyl-donor substrates and/or promotes the removal of reaction products, thereby changing the equilibrium of the ω-*O*-transacylase reaction toward increased AcylCer synthesis. Consistent with this hypothesis, our findings demonstrate that ABHD5 recruits PNPLA1 to lipid droplets. This observation indicates that the binding of PNPLA1 to ABHD5 induces a translocation of PNPLA1 to its proposed TAG substrate, providing a plausible explanation for the enhanced AcylCer biosynthesis activity in the presence of ABHD5. Whether ABHD5 also directly binds lipid substrates or recruits other proteins or factors that provide this function is still unclear and requires further investigations.

*ABHD5* mutations causing ichthyosis in humans are known to be associated with decreased levels of AcylCer (22). Here, we demonstrate that ABHD5 mutant proteins fail to stimulate PNPLA1-mediated AcylCer biosynthesis. Given that these mutants have been shown to lose their ability to localize to lipid droplets (31), our findings suggest that functional ABHD5 is indispensable for the recruitment of PNPLA1 to its acyl-donor substrate, thereby facilitating efficient esterification of ULC ω-hydroxy ceramides for the maintenance of skin integrity and barrier function.

To summarize, in our study we revealed the molecular basis of the ichthyosis pathogenesis in NLSDI patients and provided fundamental information for the development of innovative therapy strategies for the treatment of ichthyosiform skin disorders.

## Supplementary Material

Supplemental Data
