# Supplementary material for: ABHD5 stimulates PNPLA1-mediated ω-O-acylceramide biosynthesis essential for a functional skin permeability barrier
Source: J Lipid Res. 2018 Oct 25;59(12):2360–7. doi: 10.1194/jlr.M089771 (PMC6277169; doi:10.1194/jlr.M089771)
Supplement: Supplemental Data [file supp_59_12_2360__index.html]

ABHD5 Stimulates PNPLA1-mediated Omega-O-Acylceramide Biosynthesis Essential for a Functional Skin Permeability Barrier — ABHD5 stimulates PNPLA1-mediated ω-O-acylceramide biosynthesis essential for a functional skin permeability barrier — Supplemental Data 

# ABHD5 stimulates PNPLA1-mediated ω-*O*-acylceramide biosynthesis essential for a functional skin permeability barrier

## Supplemental Data

- Supplemental Data (.pdf, 332 KB) - Supplemental Data
