# Supplementary material for: ABHD5 stimulates PNPLA1-mediated ω-O-acylceramide biosynthesis essential for a functional skin permeability barrier
Source: J Lipid Res. 2018 Oct 25;59(12):2360–7. doi: 10.1194/jlr.M089771 (PMC6277169; doi:10.1194/jlr.M089771)
Supplement: Supplemental Data [file 10.1194_M089771_jlr.M089771-1.pdf]

SUPPLEMENTAL DATA:

ABHD5 Stimulates PNPLA1-mediated Omega-O-Acylceramide Biosynthesis Essential for a Functional Skin Permeability Barrier

Benedikt Kien[1], Susanne Grond[1], Guenter Haemmerle[1], Achim Lass[1,2], Thomas O. Eichmann[1,3], and Franz P. W. Radner[1,]*

[1] Institute of Molecular Biosciences, University of Graz, Graz, Austria.

[2] BioTechMed-Graz, Austria

[3] Center for Explorative Lipidomics, BioTechMed-Graz, Graz, Austria.

* To whom correspondence should be addressed: Franz P. W. Radner, Institute of Molecular Biosciences, Heinrichstraße 31/II, University of Graz, 8010 Graz, Austria. Phone: +43-316-3801921, Email: franz.radner@uni-graz.at

Supplemental Table S1. Primer pairs used for PCR amplification of respective genes. Primers were designed to create endonuclease cleavage sites (underlined) for subsequent cloning strategies.

Primer name	Sequence
***PNPLA1*-His_fw**	5'-TTCTCGAGGAAGAACAGGTGTTCAAGGG-3'
***PNPLA1*-His_rv**	5'-TTTCTAGAATCACTGCACTTTGCTGCTTG-3'
***Pnpla1*-Flag_fw**	5'-GGGAATTCACCATGGACGAACAGGTGTTCAAAG-3'
***Pnpla1*-Flag_rv**	5'-TTTCTAGAGGAGTTCTGGCCACTCACTCC-3'
***Pnpla1*-YFP_fw**	5'-TTCTCGAGCTGACGAACAGGTGTTCAAAGGAG-3'
***Pnpla1*-YFP_rv**	5'-TTGAATTCTTAGGAGTTCTGGCCACTCAC
***Atgl*-Flag_fw**	5'-TTAGATCTACCATGTTCCCGAGGGAGACC-3'
***Atgl*-Flag_rv**	5'-TTGGTACCTCGCAAGGCGGGAGGCC-3'
***Abhd5*-CFP_fw**	5'-TTCTCGAGCTAAAGCGATGGCGGCGGAG
***Abhd5*-CFP_rv**	5'-TTGGATCCTCAGTCTACTGTGTGGCAGATCTC
***ELOVL4*_fw**	5'-GGAACGCGTGGGCTCCTGGACTCGG-3'
***ELOVL4*_rv**	5'-GGTCTAGATAATCTCCTTTTGCTTTTCCATTTTTC-3'
***CYP4F22*_fw**	5'-GGACTCGAGCTGCCCATCACAGACCG-3'
***CYP4F22*_rv**	5'-GAAGCGGCCGCACCGGTGGCCCGCGGAGG-3'
***CERS3*_fw**	5'-GCGGGATCCTTTTGGACGTTTAAAGAATGGTTCTG-3'
***CERS3*_rv**	5'-GGAACTAGTATGGCCATGCTGGCCAT-3'

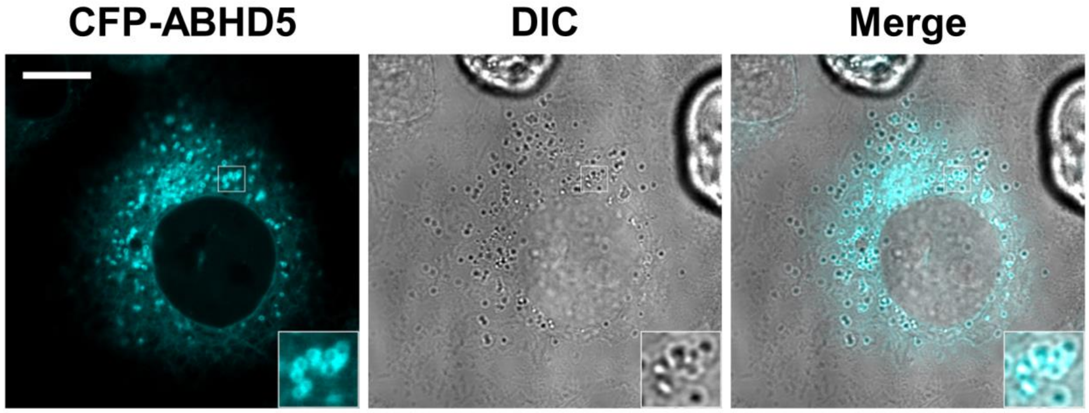

Supplemental Figure S1. ABHD5 localizes to lipid droplets in COS-7 cells. COS-7 cells were transfected with an expression vector encoding murine *Abhd5* fused to an N-terminal CFP-tag. To promote lipid droplet formation, cells were cultivated in medium supplemented with BSA-conjugated oleic acid. Intracellular localization of CFP-ABHD5 was analyzed by confocal fluorescence microscopy. Representative images are depicted. *Scale bar*, 10 µm. *Insets*, 3× zoom. Abbreviations: CFP, cyan fluorescent protein; DIC, differential interference contrast.

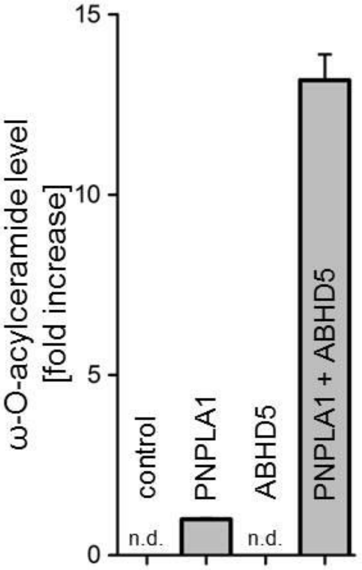

Supplemental Figure S2. ABHD5 stimulates PNPLA1-dependent AcylCer biosynthesis. Autoradiography signals of [1-^{14}C]-labeled AcylCer were obtained by exposure of developed TLC plates to a light-sensitive film. Band intensities were analyzed by densitometry using ImageJ software and normalized to cells expressing PNPLA1.

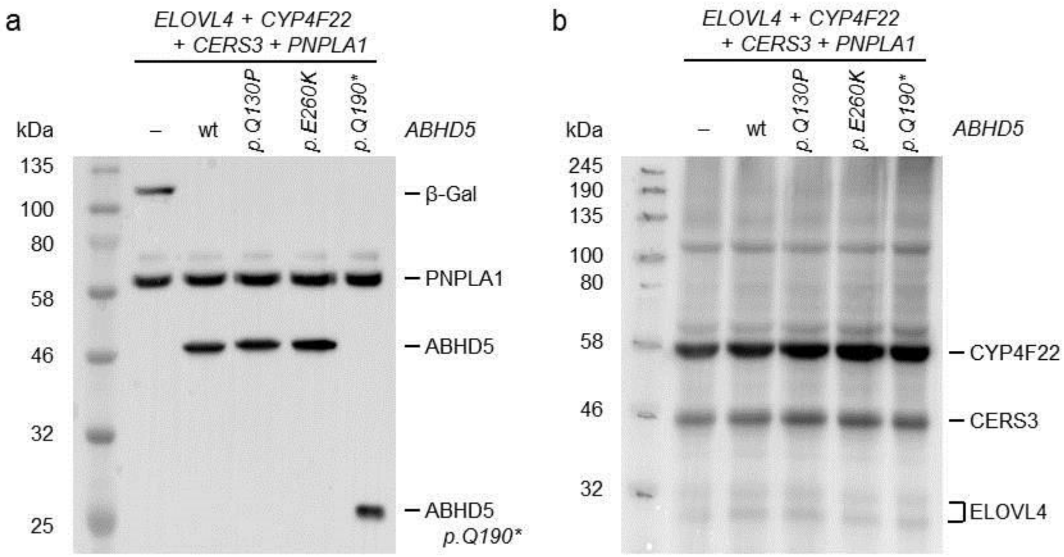

Supplemental Figure S3. Immunodetection proved similar expression levels of wild-type and mutant ABHD5 proteins in ULC ω-hydroxy ceramide-producing HEK 293T cells. HEK 293T cells were transfected with respective mammalian expression plasmids encoding human proteins. After twenty-four hours, (**a**) expression of PNPLA1 as well as mutant and wild-type ABHD5 proteins was detected using anti-Xpress® antibody, followed by (**b**) detection of ELOVL4, CYP4F22, and CERS3 expression with anti-Flag M2-HRP antibody.
